# A Novel Fixed-Time Super-Twisting Control with I&I Disturbance Observer for Uncertain Manipulators

**DOI:** 10.3390/s25216723

**Published:** 2025-11-03

**Authors:** Lin Xu, Jiahao Zhang, Chunwu Yin, Rui Dai

**Affiliations:** School of Information and Control Engineering, Xi’an University of Architecture and Technology, Xi’an 710055, China

**Keywords:** manipulators, finite time control, fixed-time control, super-twisting sliding mode control (STSMC), immersion and invariant disturbance observer

## Abstract

This paper proposes a novel fixed-time super-twisting sliding mode control (ST-SMC) strategy for uncertain robotic arm systems, aiming to address the issues of control chattering and the uncontrollable upper bound of convergence time in traditional sliding mode control algorithms. The proposed approach enhances system robustness, suppresses chattering, and ensures that the convergence time of the robotic arm can be explicitly bounded. First, a sliding surface with fixed-time convergence characteristics is constructed to guarantee that the tracking errors on this surface converge to the origin within a prescribed time. Then, an immersion and invariance (I&I) disturbance observer with exponential convergence properties is designed to estimate large, time-varying disturbances in real time, thereby compensating for system uncertainties. Based on this observer, a new super-twisting sliding mode controller is developed to drive the trajectory tracking errors toward the sliding surface within fixed time, achieving global fixed-time convergence of the tracking errors. Simulation results demonstrate that, regardless of the initial conditions, the proposed controller ensures fixed-time convergence of the tracking errors, effectively eliminates control torque chattering, and achieves a tracking error accuracy as low as 2 × 10^−9^. These results validate the proposed method’s applicability and robustness for high-precision robotic systems.

## 1. Introduction

With the rapid advancement of modern intelligent manufacturing technologies and control systems, multi-joint coupled manipulators have been increasingly integrated into high-precision motion control and industrial production processes [1,2], significantly improving production efficiency and product quality. The control accuracy and stability of the manipulator’s dynamic system directly influence the machining precision and positioning performance of high-end equipment. Therefore, for uncertain manipulator systems that are subject to multi-joint coupling, friction, joint clearance, and other nonlinearities, the design of a control algorithm with strong robustness, high tracking accuracy, and fast convergence is essential for achieving reliable performance in various engineering applications.

Traditional PID control is insufficient to meet the motion accuracy requirements of manipulators in high-precision machining applications [3]. Modern control techniques, such as backstepping control [4], active disturbance rejection control (ADRC) [5], sliding mode control (SMC) [6,7,8], neural networks [9,10], and fuzzy control [11,12], have been extensively applied to manipulator motion control to improve tracking accuracy and robustness. Among these methods, sliding mode control has attracted considerable attention due to its strong robustness, fast convergence, and simple controller structure [13]. Zhu et al. [14] combined SMC with a backstepping approach for trajectory tracking of manipulators subject to friction disturbances. Ren et al. [15] proposed an adaptive neural network–based SMC scheme to address parameter uncertainties in manipulator dynamics. Although these control strategies, including fault-tolerant control (FTC), effectively enhance system robustness and ensure asymptotic stability of the closed-loop dynamics, they do not explicitly consider the convergence rate or guarantee fixed-time convergence, which is critical for high-precision and time-sensitive applications.

The convergence rate of the manipulator’s angle tracking errors is a crucial performance index in motion control. To accelerate convergence, terminal sliding mode control (TSMC) introduces nonlinear terms into the sliding surface, thereby ensuring finite-time convergence of the tracking errors. Consequently, the convergence rate of robotic manipulators can be significantly improved. Bing et al. [16] developed a terminal sliding mode observer for uncertain robotic systems and constructed a finite-time controller using a neural network to approximate the unknown dynamics, enabling the trajectory tracking errors to converge to a small neighborhood of the origin within finite time. In another study [17], an integral terminal SMC law was proposed for robots with unknown disturbances and actuator faults, and an adaptive finite-time fault-tolerant control (FTC) algorithm was designed. The proposed control scheme not only ensured fixed-time convergence of the trajectory tracking errors but also required only the tracking error as feedback information, simplifying the controller structure.

Singularity issues are inherent in traditional terminal sliding mode control (TSMC). To overcome this limitation, the control community has proposed the nonsingular terminal sliding mode control (NTSMC) strategy. Su et al. [18] developed a TSMC method that completely eliminates singularities in uncertain robotic systems and proved the global convergence of both the sliding surface and tracking errors. Although TSMC and NTSMC can achieve finite-time convergence and effectively enhance system convergence speed, they often suffer from control chattering due to the presence of nonlinear terms and sign functions. To mitigate the chattering phenomenon, Gao [19] introduced the reaching law approach into SMC, proposing uniform-speed and exponential reaching laws. Subsequently, researchers proposed several adaptive reaching laws [20,21] to further accelerate the convergence rate. Moreover, the super-twisting second-order SMC algorithm [22,23,24] was developed to remove discontinuities in the integral term, thereby stabilizing the error convergence to zero and effectively suppressing system oscillations. Although the super-twisting algorithm provides improved robustness and smooth control action, it requires that all derivatives of the sliding variable vanish on the sliding surface, which complicates controller design and stability analysis. At present, the super-twisting algorithm has been primarily applied to single-input single-output (SISO) systems. For example, Reference [22] proposed a variable-gain super-twisting algorithm for SISO motor systems to enhance robustness while reducing the initial control torque amplitude; Reference [23] developed an adaptive super-twisting controller for wind energy conversion systems with parameter perturbations and external disturbances; and Reference [24] employed the super-twisting algorithm as a differentiator to achieve high-precision tracking in second-order SISO systems. However, extending the super-twisting algorithm to multi-input multi-output (MIMO) systems remains challenging because the state variables are coupled through correlation matrices, leading to strong variable interdependence and complex stability proofs involving high-dimensional matrix transformations. Consequently, research on MIMO super-twisting control is still in the exploratory stage. On the other hand, the conventional super-twisting sliding mode control can only ensure finite-time convergence of trajectory tracking errors, where the convergence time depends on the initial system states and control parameters. As a result, the upper bound of the convergence time cannot be predetermined, and variations in the initial conditions caused by environmental changes introduce additional uncertainty. To improve predictability, the concept of fixed-time convergence has been proposed, ensuring that the convergence time is independent of the initial states and that its upper bound remains constant under different operating conditions. This has made fixed-time control an important research topic in recent years. Nevertheless, few studies have combined the super-twisting algorithm with fixed-time convergence theory. Even in the limited existing works, the selected sliding surfaces generally lack fixed-time convergence properties, and the resulting methods cannot be regarded as genuine fixed-time super-twisting control strategies.

Sliding mode control (SMC) algorithms possess inherent robustness against system uncertainties and external disturbances. However, the conventional super-twisting algorithm can only compensate for disturbances with known upper bounds and lacks adaptability when dealing with large, time-varying uncertainties. To improve robustness under such conditions, researchers have introduced the concept of composite disturbances, which integrate both system perturbations and external disturbances, and employed fuzzy logic, neural networks, or adaptive laws to estimate and compensate for these effects. Nevertheless, fuzzy and neural network–based methods suffer from high computational complexity, while adaptive law–based estimators cannot guarantee the asymptotic convergence of the composite disturbance estimation error to zero.

Based on the above analysis, the existing super-twisting control algorithms exhibit the following limitations:

(1) The conventional super-twisting control algorithm ensures finite-time convergence; however, the maximum convergence time depends on the initial states and control parameters, making it uncontrollable and unpredictable. Although Tran et al. [25] proposed a fixed-time super-twisting algorithm whose upper bound of convergence time depends only on control parameters, this method is applicable only to single-input single-output (SISO) systems and cannot be directly extended to multi-input multi-output (MIMO) cases.
(2) Fuzzy logic, neural network, and adaptive control approaches are commonly employed to estimate composite disturbances in complex control systems. However, these methods often suffer from non-vanishing estimation errors, that is, the estimation error of composite disturbances does not asymptotically converge to zero.
(3) The majority of existing sliding mode surfaces exhibit asymptotic or finite-time convergence characteristics, lacking fixed-time convergence properties. Sliding mode controllers designed under the fixed-time convergence framework typically ensure that only the sliding surface reaches zero within a fixed time, without guaranteeing that the tracking error confined to the surface also converges to zero within the same fixed time. Consequently, the system tracking error cannot be guaranteed to vanish absolutely within a fixed time.

Therefore, to fully exploit the advantages of the super-twisting second-order sliding mode control (ST-SMC) in suppressing system oscillations, this paper proposes an enhanced fixed-time super-twisting control framework that addresses the aforementioned limitations from the following perspectives:

(1) The fixed-time super-twisting algorithm for single-input single-output (SISO) systems described in [25] is extended to multi-input multi-output (MIMO) systems using a fixed-time convergence approach. A fixed-time adaptive super-twisting sliding mode controller is developed for the MIMO robotic arm dynamic system with parameter perturbations. The proposed controller enables the end-effector to achieve fast and accurate trajectory tracking under complex operating conditions while effectively suppressing high-frequency chattering.
(2) An Immersion and Invariance (I&I) disturbance observer is designed to estimate external composite disturbances with minimal estimation error. The observer features a simple structure and guarantees exponential convergence of the estimation error to zero, thereby overcoming the non-convergence issues of conventional composite disturbance estimators and mitigating the impact of external disturbances on trajectory tracking accuracy.
(3) A sliding mode surface with fixed-time convergence characteristics is constructed, ensuring that tracking errors on the surface converge to zero within a fixed time. Consequently, the trajectory tracking error of the overall system achieves global fixed-time convergence, further enhancing the precision and robustness of the control performance.

## 2. Problem Formulation and Model Description

**Figure 1:**
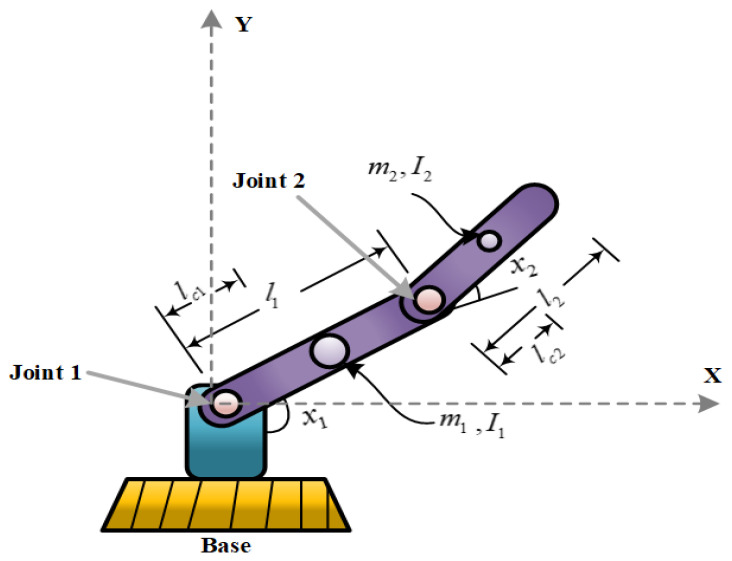
Schematic diagram of the 2DOF robotic manipulator.

Consider a two-degree-of-freedom (DOF) robotic manipulator as depicted in Figure 1; it is considered that the model contains external disturbance and parameter perturbation. The system dynamics can be expressed as

(1) $$M(x(t))\ddot{x}(t)+N(x(t),\dot{x}(t))\dot{x}(t)+G(x(t))=u(t)+d(t)$$

where $x(t),\;\dot{x}(t),\;\ddot{x}(t)$ denote the angle, angular velocity, and angular acceleration of the manipulator, respectively; u(t) denotes the input variable; d(t) denotes external disturbance termed as disturbance herein; M(x(t)) denotes inertia matrix, $N(x(t),\dot{x}(t))$ denotes centripetal force matrix; and G(x(t)) denotes gravity vector.

The inertial matrix, centripetal force matrix, and gravity matrix of the robot are perturbed by measurement errors and wear or load during use, namely $M(x(t))=M_{0}(x(t))+\Delta M(x(t))$, $G(x(t))=G_{0}(x(t))+\Delta G(x(t))$, $N(x(t))=N_{0}(x(t))+\Delta N(x(t))$. $M_{0}(x(t)),\;N_{0}(x(t)),$ and $G_{0}(x(t))$ denote the nominal matrix calculated based on the measured mass and length of the manipulator. ΔM(x(t)), ΔN(x(t)), ΔG(x(t)) denote parameter perturbation occasioned by measurement error and wear or load during use.

Denoting $x_{1}(t)=x(t),\;x_{2}(t)=\dot{x}(t)$, $\eta (t)=M_{0}^{-1}(t)\left(-\Delta N(t)x_{2}(t)-\Delta G(t)d(t)\right)$, the dynamic Equation (1) of the manipulator can be converted to

(2) $$\left\{\begin{array}{l} {\dot{x}}_{1}(t)=x_{2}(t)\\ {\dot{x}}_{2}(t)=-M_{0}^{-1}(t)N_{0}(t)x_{2}(t)-M_{0}^{-1}(t)G_{0}(t)+M_{0}^{-1}(t)u(t)+\eta (t)\\ y(t)=x_{1}(t) \end{array}\right.$$

The control objective is to design a sliding mode controller; thus, the state variable $x_{1}(t)$ can accurately track the given trajectory $x_{d}(t)$ within a fixed time.

**Lemma 1**  [25]**.** *Generalized super-twisting SISO systems with disturbance are expressed as*

(3) $$\left\{\begin{array}{l} {\dot{x}}_{1}(t)=-k_{1}f_{1}(x_{1}(t))+x_{2}(t)+d_{1}(t)\\ {\dot{x}}_{2}(t)=-k_{2}f_{2}(x_{1}(t))+d_{2}(t) \end{array}\right.$$

*where stat* $x_{1}(t),\;x_{2}(t)\in R,\;$ *parameter* $k_{1},\;k_{2}>0,\;$ *and* $d_{1}(t),\;d_{2}(t)\;$ *are external disturbance, and the functions* $f_{1}(x_{1}(t)),\;$ $f_{2}(x_{1}(t))\;$ *are defined as*

(4) $$\left\{\begin{array}{cc} f_{1}(x_{1}(t))= & \gamma_{1}{\mathrm{sig}}^{\frac{1}{2}}(x_{1}(t))+\gamma_{2}{\mathrm{sig}}^{\alpha_{1}}(x_{1}(t))+\gamma_{3}{\mathrm{sig}}^{\alpha_{2}}(x_{1}(t))\\ f_{2}(x_{1}(t))= & \frac{1}{2}\gamma_{1}^{2}{\mathrm{sig}}^{0}(x_{1}(t))+(\frac{1}{2}+\alpha_{1})\gamma_{1}\gamma_{2}{\mathrm{sig}}^{\alpha_{1}-\frac{1}{2}}(x_{1}(t))\\ & +\alpha_{1}\gamma_{2}^{2}{\mathrm{sig}}^{2\alpha_{1}-1}(x_{1}(t))+(\frac{1}{2}+\alpha_{2})\gamma_{1}\gamma_{3}{\mathrm{sig}}^{\alpha_{2}-\frac{1}{2}}(x_{1}(t))\\ & +\alpha_{2}\gamma_{3}^{2}{\mathrm{sig}}^{2\alpha_{2}-1}(x_{1}(t))+(\alpha_{1}+\alpha_{2})\gamma_{2}\gamma_{3}{\mathrm{sig}}^{\alpha_{1}+\alpha_{2}-1}(x_{1}(t)) \end{array}\right.$$

*where parameter *$\gamma_{1},\;\gamma_{2},\;\gamma_{3}\geq 0,\;\frac{1}{2}\leq \alpha_{1}\leq 1<\alpha_{2}$. ${\mathrm{sig}}^{\alpha_{1}}(x_{1}(t))=|x_{1}(t){|}^{\alpha_{1}}\mathrm{sign}(x_{1}(t)),\;$*and *$\mathrm{sign}(x_{1}(t))$* is a sign function of *$x_{1}(t),\;$$\left|x_{1}(t)\right|$* is the absolute value of *$x_{1}(t)$.

(1) Disturbances $d_{1}(t),\;d_{2}(t)$ satisfy $|d_{i}(t)|\leq \delta_{i}|f_{i}(x_{1}(t))|,\;\;i=1,2$, where $\delta_{i}\geq 0$
(2) Let $\xi (t)={\left[\xi_{1}(t),\xi_{2}(t)\right]}^{T}={\left[f_{1}(x_{1}(t)),x_{2}(t)\right]}^{T}$, $A=\left[\begin{array}{cc} -k_{1} & 1\\ -k_{2} & 0 \end{array}\right],\;B=\left[\begin{array}{cc} 1 & 0\\ 0 & 1 \end{array}\right],\;H=\left[\begin{array}{c} 1\\ 0 \end{array}\right]$. There exists a positive definite matrix Θ and any positive number ε, such that the symmetric positive definite matrix P satisfies

(5) $$A^{Τ}P+PA+\varepsilon P+H+PB\Theta^{-1}B^{Τ}P\leq 0$$

If the Lyapunov function is defined as

(6) $$V(t)=\xi^{Τ}(t)P\xi (t)+\frac{k_{2}\varepsilon }{2}|f_{1}(x_{1}(t)){|}^{2}-{\mathrm{sig}}^{\frac{1}{\alpha_{2}}}(f_{1}(x_{1}(t))){\mathrm{sig}}^{\frac{2\alpha_{2}-1}{\alpha_{2}}}(x_{2}(t))+\frac{\varepsilon }{2}x_{2}^{2}(t)$$

the states in system (3) converge to the origin in fixed time. The inequality given in (7) holds true.

(7) $$\dot{V}(t)\leq -c_{0}V^{\frac{1}{2}}(t)-c_{1}V^{\frac{3\alpha_{1}-1}{2\alpha_{1}}}(t)-c_{2}V^{\frac{3\alpha_{2}-1}{2\alpha_{2}}}(t)$$

**Lemma 2**  [26]**.** *For variable* $x_{i}\in R,\;i=1,2,\ldots ,n,\;$ *the inequalities (8) and (9) hold true.*

(8) $${\left(\sum_{i=1}^{n}\left|x_{i}\right|\right)}^{l}\leq \sum_{i=1}^{n}|x_{i}{|}^{l}\;\mathrm{where}\;l\in [0,1]$$

(9) $$n^{1-l}{\left(\sum_{i=1}^{n}\left|x_{i}\right|\right)}^{l}\leq \sum_{i=1}^{n}|x_{i}{|}^{l}\;\mathrm{where}\;l\in [1,\infty ]$$

**Lemma 3**  [27]**.** *If there are continuously differentiable positive definite functions* V(x),  *positive real number* $\mu_{1},\;\mu_{2},\alpha \in [1,\infty ),\;$ *and parameter* β∈(0,1),  *the inequality (10) holds.*

(10) $$\dot{V}(x)\leq -\mu_{1}V^{\alpha }(x)-\mu_{2}V^{\beta }(x)$$

Subsequently, the system is globally stable with fixed time, and the convergence time $T_{s}$ satisfies (11)

(11) $$T_{s}\leq \frac{1}{\mu_{1}(\alpha -1)}+\frac{1}{\mu_{2}(1-\beta )}$$

Representing the trajectory tracking error e(t) and its derivative $\dot{e}(t)$ of the manipulator are

(12) $$\begin{array}{l} e(t)=x_{1}(t)-x_{d}(t)\\ \dot{e}(t)=x_{2}(t)-{\dot{x}}_{d}(t) \end{array}$$

## 3. Design of the Fixed-Time Super-Twisting Sliding Mode Controller

**Step 1: Design of the fast terminal sliding mode surface**

The fast terminal sliding mode (TSM) surface of fixed-time convergence based on tracking error is constructed as

(13) $$S(t)=\dot{e}(t)+k_{e1}{\mathrm{sig}}^{\frac{p_{1}}{q_{1}}}(e(t))+k_{e2}{\mathrm{sig}}^{\frac{q_{2}}{p_{2}}}(e(t))$$

where the positive integer parameters $p_{i}>q_{i}>0$, and $k_{e1},\;k_{e2}>0$ are the control gain of the sliding variable.

**Figure 2:**
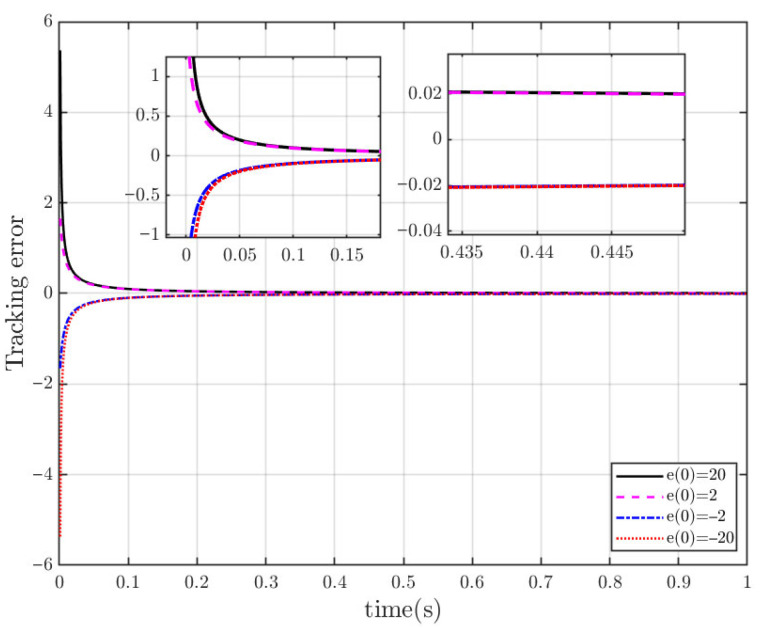
Trajectory tracking error for the different initial values.

To confirm the fixed-time convergence characteristic of tracking error within the sliding surface (13), assume that the tracking error reaches the sliding mode surface (designated as S=0), and set the corresponding initial values of tracking error as e(0)=20, e(0)=2, e(0)=−2, e(0)=−20. The numerical simulation is presented in Figure 2, which indicates that the actual convergence times are the same under different tracking error initial values; thus, the convergence time is not affected by the initial value of tracking error.

**Step 2: Design of I&I Disturbance Observer**

The principle of Immersion and Invariance (I&I) is an emerging control strategy for nonlinear systems developed by Astolfi and Ortega in 2003 [28] based on the concept of differential geometry, where a one-dimensional target system that is asymptotically stable at the origin is immersed into a higher-dimensional original system. I&I control strategies do not need to construct Lyapunov functions, but only need to prove that the target system can be immersed into another object and into the state variables in an invariant manifold [29,30]. The characteristics of the target function and the object are asymptotically consistent.

Based on the I&I theory, the I&I disturbance observer is designed for the sub-system

(14) $${\dot{x}}_{2}(t)=-M_{0}^{-1}(t)N_{0}(t)x_{2}(t)-M_{0}^{-1}(t)G_{0}(t)+M_{0}^{-1}(t)u(t)+\eta (t)$$

To observe the unknown external disturbance vector η(t), let the upper bound of η(t) be $\eta_{\mathrm{upper}}(t)$, and the estimated value of $\eta_{\mathrm{upper}}(t)$ be $\hat{\eta }(t)$. Thus, the system is constructed as expressed in (15):

(15) $$\left\{\begin{array}{l} \hat{\eta }(t)=z_{I\&I}(t)+\Phi (x_{2}(t))\\ {\dot{z}}_{I\&I}(t)=\omega (x_{2}(t),z_{I\&I}(t)) \end{array}\right.$$

where $z_{I\&I}(t)$ denotes the auxiliary parameter. The adjustment function $\Phi (x_{2}(t))$ and the parameter adaptive law function $\omega (x_{2}(t),z_{I\&I}(t))$ are the functions to be designed. A one-dimensional manifold is defined on the extended state space $\left(x_{2}(t),z_{I\&I}(t)\right)$ as $ℵ=\{(x_{2}(t),z_{I\&I}(t))\in R^{2n}|\eta_{\mathrm{upper}}(t)-z_{I\&I}(t)-\Phi (x_{2}(t))=0\}$

Defining manifold coordinates, the estimated error of external disturbance is written as

(16) $$\tilde{\eta }(t)=\eta_{\mathrm{upper}}(t)-\hat{\eta }(t)=\eta_{\mathrm{upper}}(t)-z_{I\&I}(t)-\Phi (x_{2}(t))$$

Taking the derivative of (16), we obtain

(17) $$\begin{array}{cc} \dot{\tilde{\eta }}(t) & ={\dot{\eta }}_{\mathrm{upper}}(t)-{\dot{z}}_{I\&I}(t)-\dot{\Phi }(x_{2}(t))\\ & ={\dot{\eta }}_{\mathrm{upper}}(t)-\omega (x_{2}(t),z_{I\&I}(t))-\frac{d\Phi }{dx_{2}}(-M_{0}^{-1}(t)N_{0}(t)x_{2}(t)-M_{0}^{-1}(t)G_{0}(t)+M_{0}^{-1}(t)u(t)+\eta (t)) \end{array}$$

To make the manifold ℵ systematic, the parameter adaptive law function is designed as

(18) $$\omega (x_{2}(t),z_{I\&I}(t))=-\frac{d\Phi }{dx_{2}}(-M_{0}^{-1}(t)N_{0}(t)x_{2}(t)-M_{0}^{-1}(t)G_{0}(t)+M_{0}^{-1}(t)u(t)+\hat{\eta }(t))$$

Therefore,

(19) $$\dot{\tilde{\eta }}(t)={\dot{\eta }}_{\mathrm{upper}}(t)-\frac{d\Phi }{dx_{2}}\tilde{\eta }(t)$$

The upper bound $\eta_{\mathrm{upper}}(t)$ is a constant; therefore, ${\dot{\eta }}_{\mathrm{upper}}(t)=0$. If the adjustment function is designed as $\Phi (x_{2}(t))=k_{I\&I}x_{2}(t)$, we obtain

(20) $$\dot{\tilde{\eta }}(t)=-k_{I\&I}\tilde{\eta }(t)$$

Equation (20) indicates that for any positive number $k_{I\&I}$, the disturbance tracking error $\tilde{\eta }(t)$ converges exponentially to zero, and the convergence rate is determined by $k_{I\&I}$: the accurate estimation of external disturbance can be achieved by adopting (15).

Based on the preceding analysis, the I&I disturbance observer is

(21) $$\left\{\begin{array}{l} \hat{\eta }(t)=z_{I\&I}(t)+k_{I\&I}x_{2}(t)\\ {\dot{z}}_{I\&I}(t)=k_{I\&I}(M_{0}^{-1}(t)N_{0}(t)x_{2}(t)+M_{0}^{-1}(t)G_{0}(t)-M_{0}^{-1}(t)u(t)-\hat{\eta }(t)) \end{array}\right.$$

The vital purpose of designing an adaptive estimation method based on the I&I scheme is to make the error manifold convergent and to precisely estimate this invariable, which is achieved by adjusting parameters such as $\omega (x_{2}(t),z_{I\&I}(t))$ and the regulating function $\Phi (x_{2}(t))$. These parameters are responsible for ensuring the asymptotic convergence pertaining to the disturbance estimation of the real value. Moreover, the traditional adaptive laws utilize the equivalence principle, adjust this function $\Phi (x_{2}(t))$, and can transform the dynamic modification method of disturbance estimation from a single integral function to a proportional integral function. This transformation successfully advances the freedom of the dynamic modification method of disturbance estimation and leads to accurate estimation.

**Step 3: Design of the controller**

Design a novel super-twisting sliding mode control based on an I&I disturbance observer for uncertain robot systems is

(22) $$\left\{\begin{array}{cc} u(t) & =N_{0}(t)x_{2}(t)-M_{0}(t)\Psi (t)\dot{e}(t)+G_{0}(t)+M_{0}(t)\left({\ddot{x}}_{d}(t)-k_{s1}f_{1}(S(t))+\chi (t)-\hat{\eta }(t)\right)\\ \dot{\chi }(t) & =-k_{s2}f_{2}(S(t)) \end{array}\right.$$

where χ(t) denotes an auxiliary variable. The parameters $k_{s1},\;k_{s2}$ are positive definite diagonal matrices. The functions $f_{1}(S(t))$, $f_{2}(S(t))$, Ψ(t) are

(23) $$f_{1}(S(t))=\gamma_{1}{\mathrm{sig}}^{\frac{1}{2}}(S(t))+\gamma_{2}{\mathrm{sig}}^{\alpha_{1}}(S(t))+\gamma_{3}{\mathrm{sig}}^{\alpha_{2}}(S(t))$$

(24) $$\begin{array}{cc} f_{2}(S(t)) & =\frac{1}{2}\gamma_{1}^{2}{\mathrm{sig}}^{0}(S(t))+(\frac{1}{2}+\alpha_{1})\gamma_{1}\gamma_{2}{\mathrm{sig}}^{\alpha_{1}-\frac{1}{2}}(S(t))+\alpha_{1}\gamma_{2}^{2}{\mathrm{sig}}^{2\alpha_{1}-1}(S(t))\\ & +(\frac{1}{2}+\alpha_{2})\gamma_{1}\gamma_{3}{\mathrm{sig}}^{\alpha_{2}-\frac{1}{2}}(S(t))+\alpha_{2}\gamma_{3}^{2}{\mathrm{sig}}^{2\alpha_{2}-1}(S(t))+(\alpha_{1}+\alpha_{2})\gamma_{2}\gamma_{3}{\mathrm{sig}}^{\alpha_{1}+\alpha_{2}-1}(S(t)) \end{array}$$

(25) $$\Psi (t)=\left\{\begin{array}{cc} k_{e1}\frac{p_{1}}{q_{1}}{\mathrm{sig}}^{\frac{p_{1}}{q_{1}}-1}(e(t))+k_{e2}\frac{q_{2}}{p_{2}}{\mathrm{sig}}^{\frac{q_{2}}{p_{2}}-1}(e(t)) & \min|e(t)|\geq \varepsilon \\ k_{e1}\frac{p_{1}}{q_{1}}{\mathrm{sig}}^{\frac{p_{1}}{q_{1}}-1}(e(t)) & \min|e(t)|<\varepsilon \end{array}\right.$$

where parameter $\gamma_{1},\;\gamma_{2},\;\gamma_{3}\geq 0,\;\frac{1}{2}\leq \alpha_{1}\leq 1<\alpha_{2}$. ${\mathrm{sig}}^{\alpha_{1}}(x_{1}(t))=|x_{1}(t){|}^{\alpha_{1}}\mathrm{sign}(x_{1}(t))$, ε is a small positive.

## 4. Stability Analysis

**Theorem 1.** 
*For the fast terminal sliding mode surface (13), if the sliding mode surface satisfied* S(t)=0,  *then, the tracking error* e(t) *is convergent in fixed time.*

**Proof.** 
If S(t)=0, then, $\dot{e}(t)=-k_{e1}{\mathrm{sig}}^{\frac{p_{1}}{q_{1}}}(e(t))-k_{e2}{\mathrm{sig}}^{\frac{q_{2}}{p_{2}}}(e(t))$. Defined Lyapunov function as

(26) $$V_{e}(t)=\frac{1}{2}e^{Τ}(t)e(t)$$

Then

(27) $$\begin{array}{cc} \dot{V}(t) & =e^{Τ}(t)\dot{e}(t)\\ & =e^{Τ}(t)\left(-k_{e1}{\mathrm{sig}}^{\frac{p_{1}}{q_{1}}}(e(t))-k_{e2}{\mathrm{sig}}^{\frac{q_{2}}{p_{2}}}(e(t))\right)\\ & \leq -2^{\frac{q_{1}+p_{1}}{2q_{1}}}k_{e1}{\left(\frac{1}{2}e^{Τ}(t)e(t)\right)}^{\frac{q_{1}+p_{1}}{2q_{1}}}-2^{\frac{q_{2}+p_{2}}{2p_{2}}}k_{e2}{\left(\frac{1}{2}e^{Τ}(t)e(t)\right)}^{\frac{q_{2}+p_{2}}{2p_{2}}}\\ & \leq -2^{\frac{q_{1}+p_{1}}{2q_{1}}}k_{e1}V_{e}^{\frac{q_{1}+p_{1}}{2q_{1}}}(t)-2^{\frac{q_{2}+p_{2}}{2p_{2}}}k_{e2}V_{e}^{\frac{q_{2}+p_{2}}{2p_{2}}} \end{array}$$

According to Lemma 3, it can be obtained that the tracking error e(t) is convergent in fixed time. □

**Theorem 2.** 
* For the manipulator dynamic system (2), under the action of the I&I disturbance observer (21) and the following generalized STSMC (22), then the system (2) is convergent in fixed-time, i.e., the manipulator angle can converge to the desired trajectory in a fixed time.*

**Proof.**  According to the principle of sliding mode control, the convergence of trajectory tracking errors consists of an approaching phase and a sliding phase. In the approaching phase, the tracking errors at any initial position converge to the sliding mode surface, which requires the sliding mode surface to converge to zero within a fixed time. In the sliding phase, the tracking errors within the sliding mode surface converge to zero within a fixed time. □

**Step 1: Prove that the sliding mode surface converges to zero within a fixed time.**

The derivative of the fast terminal sliding mode surface with fixed-time convergence (13) is

(28) $$\begin{array}{cc} \dot{S}(t) & =\ddot{e}(t)+\left(k_{e1}\frac{p_{1}}{q_{1}}{\mathrm{sig}}^{\frac{p_{1}}{q_{1}}-1}(e(t))+k_{e2}\frac{q_{2}}{p_{2}}{\mathrm{sig}}^{\frac{q_{2}}{p_{2}}-1}(e(t))\right)\dot{e}(t)\\ & =-M_{0}^{-1}(t)N_{0}(t)x_{2}(t)-M_{0}^{-1}(t)G_{0}(t)+M_{0}^{-1}(t)u(t)-{\ddot{x}}_{d}(t)\\ & +\left(k_{e1}\frac{p_{1}}{q_{1}}{\mathrm{sig}}^{\frac{p_{1}}{q_{1}}-1}(e(t))+k_{e2}\frac{q_{2}}{p_{2}}{\mathrm{sig}}^{\frac{q_{2}}{p_{2}}-1}(e(t))\right)\dot{e}(t)+\eta (t) \end{array}$$

By substituting (22) into (28), the generalized super-twisting sliding mode reaching law can be expressed as

(29) $$\left\{\begin{array}{l} \dot{S}(t)=-k_{s1}f_{1}(S(t))+\chi (t)+\tilde{\eta }(t)\\ \dot{\chi }(t)=-k_{s2}f_{2}(S(t)) \end{array}\right.$$

The structure of (29) is similar to that of the system in Lemma 1. Therefore, according to Lemma 1, for each subsystem in the generalized super-twisting sliding mode reaching law, the Lyapunov functions $V_{s1}(t),\;V_{s2}(t),\;V_{s3}(t)$, whose design form is similar to (6), are designed as

(30) $${\dot{V}}_{\mathrm{si}}(t)\leq -c_{\mathrm{si}0}V_{\mathrm{si}}^{\frac{1}{2}}(t)-c_{\mathrm{si}1}V_{\mathrm{si}}^{\frac{3\alpha_{1}-1}{2\alpha_{1}}}(t)-c_{\mathrm{si}2}V_{\mathrm{si}}^{\frac{3\alpha_{2}-1}{2\alpha_{2}}}(t)$$

To analyze the stability of system (29), the Lyapunov function given in (27) is constructed

(31) $$V_{S}(t)=\sum_{i=1}^{3}V_{\mathrm{si}}(t)$$

According to (26) and Lemma 2,

(32) $${\dot{V}}_{S}(t)=\sum_{i=1}^{3}{\dot{V}}_{\mathrm{si}}(t)\leq -c_{s0}V_{\mathrm{si}}^{\frac{1}{2}}(t)-c_{s1}V_{\mathrm{si}}^{\frac{3\alpha_{1}-1}{2\alpha_{1}}}(t)-3^{\frac{2\alpha_{2}}{\alpha_{2}-1}}c_{s2}V_{\mathrm{si}}^{\frac{3\alpha_{2}-1}{2\alpha_{2}}}(t)$$

where $c_{s0}=\underset{i=1,2,3}{\min}\{c_{\mathrm{si}0}\},\;c_{s1}=\underset{i=1,2,3}{\min}\{c_{\mathrm{si}1}\},\;c_{s2}=\underset{i=1,2,3}{\min}\{c_{\mathrm{si}2}\}$; subsequently, the sliding mode surface S(t) in the generalized super-twisting sliding mode reaching law converges to zero in fixed time.

**Step 2: Prove that the tracking errors within the sliding mode surface converge to zero within a fixed time.**

At the condition where the sliding variable satisfies S(t)=0, according to Theorem 1, the tracking error e(t) can exhibit apparent convergence and approaches to zero within a fixed time.

## 5. Numerical Simulation Analysis

Numerical simulations have been conducted in the MATLAB R2018a/Simulink environment, and a laptop with the following specifications was utilized: 11th Gen Intel^®^ Core™ i5-1135G7 @ 2.40 GHz, 2.42 GHz

### 5.1. Control and Simulation Parameters

In this section, a dynamic equation of a two-DOF robotic manipulator is considered in the simulation, and the designed fixed-time STSMC algorithm is numerically simulated. The simulation scenario includes the following: two joint configurations are $\sin\left(3t\right)$ and $cos\left(3t\right)$, initial value of the desired trajectory is $x_{d}={\left[0,1\right]}^{T}$, initial angle and angular velocity of mechanical arm is $\left[{x\left(0\right)=\left[1.5,0.2\right]}^{T},\dot{x}\left(0\right)\right]=[0$.1, 0.1]^T^ and external disturbance is $d(t)={\left[3sin(0.1t),1-e^{-0.1t}\right]}^{T}$. The simulation time was set as 5 s, and the relevant parameters of the manipulator are illustrated in Table 1 [31] as follows:

Controller parameters are set as Table 2:

### 5.2. Effectiveness Analysis

**Figure 3:**
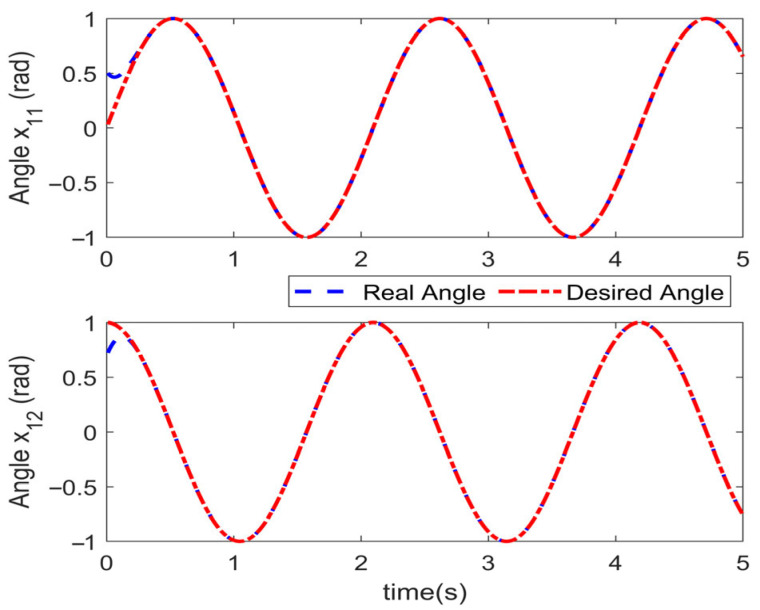
Angle tracking performance.

**Figure 4:**
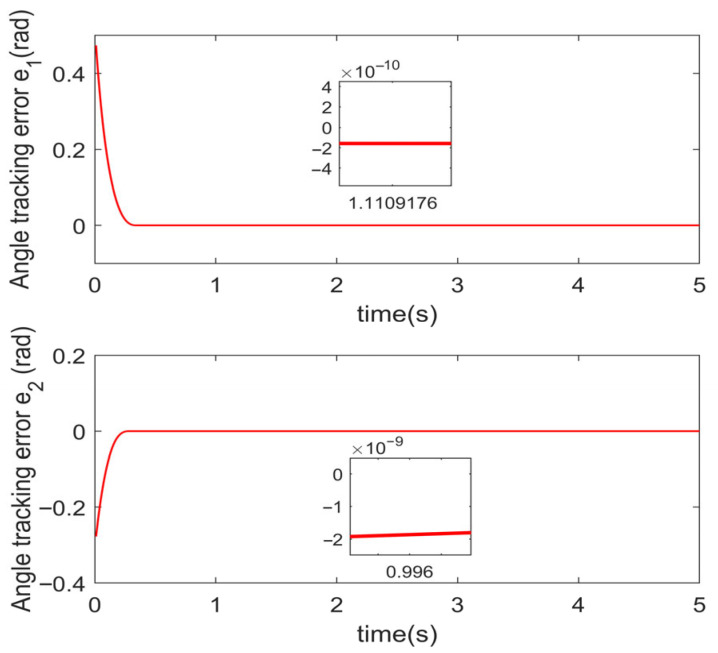
Angle tracking error.

**Figure 5:**
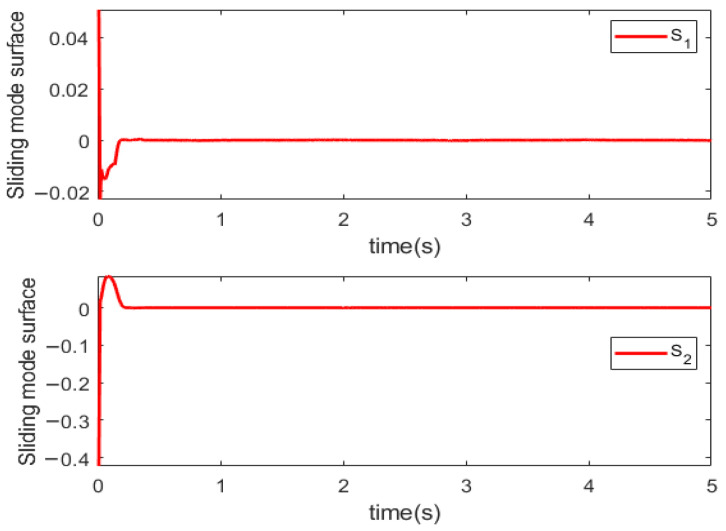
Sliding mode surface.

**Figure 6:**
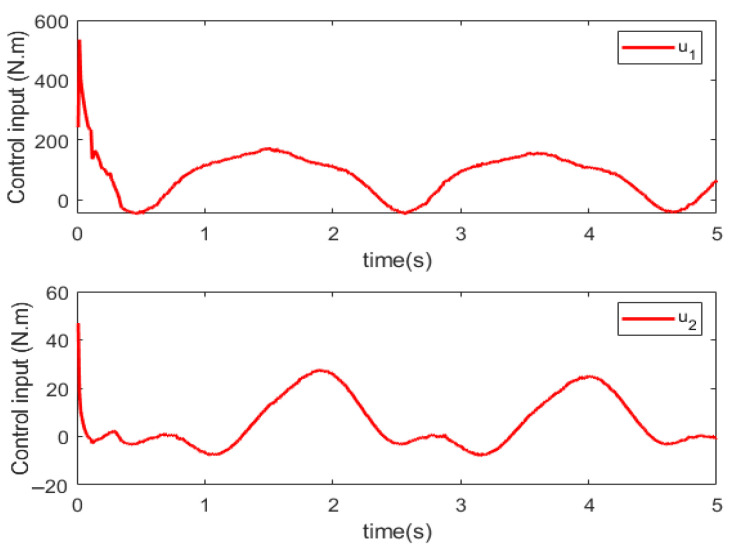
Control input.

Simulations are conducted to characterize the control performance and to quantify the efficacy of the proposed control strategy. Results are depicted in Figure 3, Figure 4, Figure 5 and Figure 6. Figure 3 demonstrates the end position tracking pertaining to the desired trajectory of the manipulator, whereas the corresponding trajectory error is illustrated in Figure 4. Figure 5 depicts the fixed-time convergence of the sliding mode surface, whereas the corresponding control effort of the STSMC is illustrated in Figure 6.

Figure 3 and Figure 4 indicate that under the simulation conditions of external disturbance and the time-varying internal parameter perturbation of the manipulator, the proposed fixed-time super-twisting sliding mode controller permits the end position of the manipulator to accurately track the desired trajectory within 0.4 s. The accuracy of the trajectory tracking error attains $2\times 10^{-10}$ and $2\times 10^{-9}$ in both cases, which indicates that the proposed controller not only demonstrates an excellent performance in regard to fast convergence speed and high tracking accuracy, but also exhibits strong robustness to external disturbance and internal time-varying parameter perturbation.

Figure 6 indicates that the sliding mode surface closely approximates zero within 0.4 s and subsequently remains unchanged. Thus, the sliding mode surface rapidly converges to zero within a fixed time, indicating that the proposed control can ensure rapid convergence of the sliding variable to zero. Figure 6 indicates that when the initial trajectory tracking error is large, the control input torque is large, $u_{1}$ attaining 584 Nm; contrastingly, the control torque $u_{2}$ is relatively small, attaining a maximum value of 50 Nm. After the system attains a stable state, the control torque gradually decreases. In the whole control process, the control torque of the manipulator changes gradually with no oscillations, thereby indicating that the proposed controller effectively reduces the inherent oscillatory phenomenon in the sliding mode control algorithm.

### 5.3. Comparative Analysis of Different Controllers

To compare and analyze the advantages of the fixed-time convergent STSMC algorithm (FixTC) in regard to convergence speed and convergence accuracy, the traditional asymptotically stable PID controller and the adaptive terminal sliding mode controller (FiniteTC) with finite-time convergence characteristics are selected for comparison in simulation.

The PID-based control law has been expressed as $u(t)=k_{p}e+k_{d}\dot{e}+k_{i}\int_{0}^{t}ed\tau$.

The adaptive terminal sliding mode control law (FiniteTC) is expressed as

$$\left\{\begin{array}{l} S=\dot{e}+k_{e2}{\mathrm{sig}}^{\frac{q_{2}}{p_{2}}}(e)\\ u(t)=Nx_{2}+G-M\left(\hat{\eta }-{\ddot{x}}_{d}+k_{e2}\frac{q_{2}}{p_{2}}{\mathrm{sig}}^{\frac{q_{2}}{p_{2}}-1}(e)\dot{e}+k_{s1}S\right)\\ \dot{\hat{\eta }}=Q^{-1}S \end{array}\right.$$

PID controller parameters are set as $k_{p}=5000,\;k_{d}=500,\;k_{i}=10$. The parameters of the adaptive TSMC (FiniteTC) with finite-time convergence are set as $k_{e2}=5;\;p_{2}=5,\;q_{2}=3;\;$ $k_{s1}=\mathrm{diag}([300,300]);\;Q=\mathrm{diag}([11,11])$

**Figure 7:**
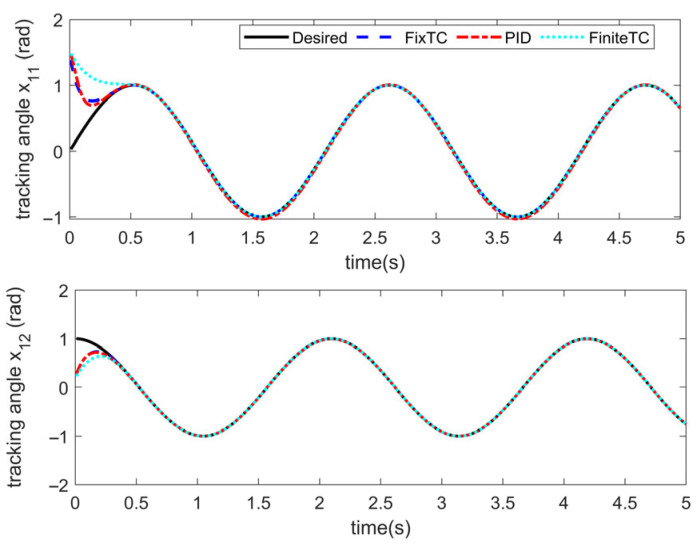
Angle tracking.

**Figure 8:**
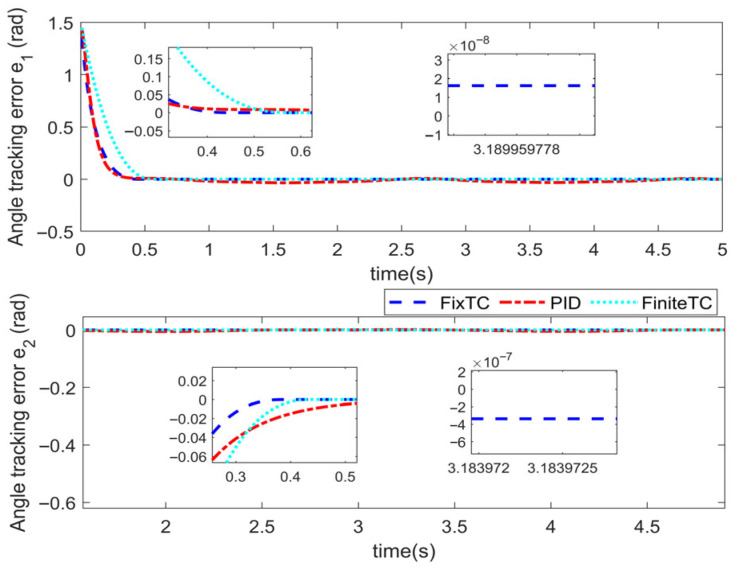
Angle tracking error.

**Figure 9:**
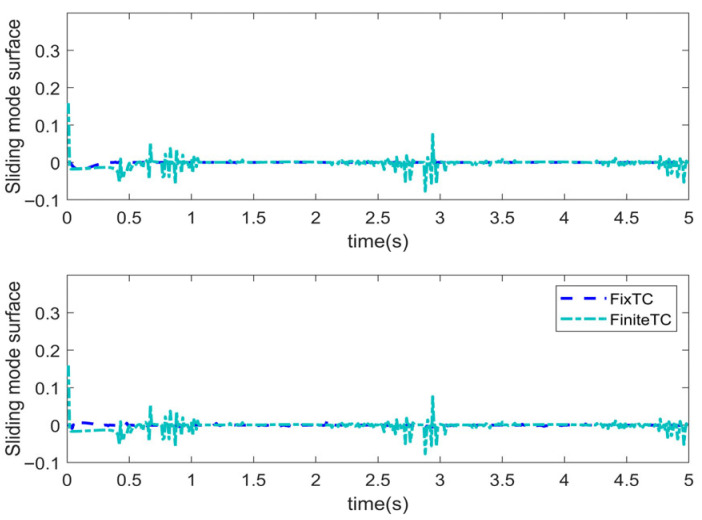
Sliding mode surface.

**Figure 10:**
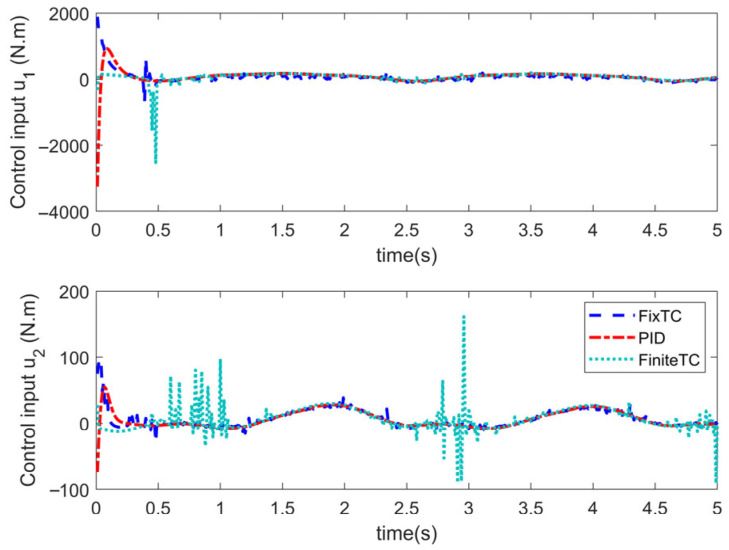
Control input.

The parameter settings of the STSMC algorithm (FixTC) designed herein are the same as those mentioned in Section 5.1. The initial angle and angular velocity of the manipulator is ${x\left(0\right)=\left[1.5,0.2\right]}^{T},\;\dot{x}\left(0\right)=[$0.1, 0.1]^T^, and the results of the numerical simulation are depicted in Figure 7, Figure 8, Figure 9 and Figure 10.

Figure 7 and Figure 8 indicate the following: all three controllers ensure that the end position of the manipulator can meet the desired trajectory within 0.6 s. Although the convergence time of the three controllers is similar, the convergence time of the proposed STSMC algorithm is relatively smaller, thus indicating a faster response. From the perspective of accuracy in trajectory tracking, as depicted in Figure 7, the tracking error accuracy of the PID controller is $4\times 10^{-2}$ and $1\times 10^{-3}$, the tracking error accuracy of the adaptive terminal sliding mode controller (FiniteTC) is $2\times 10^{-7}$ and $2\times 10^{-5}$, and the tracking error accuracy of the proposed FixTC control algorithm is $2\times 10^{-10}$ and $2\times 10^{-9}$. The error curve of trajectory tracking under the PID controller is not completely stable as can be observed in Figure 8. In summary, the proposed controller exhibits faster convergence speed and higher trajectory tracking accuracy and is more consistent with the control applications requiring high-precision trajectory tracking.

Figure 9 illustrates the variation trend pertaining to the sliding variable of FiniteTC and FixTC. It is indicated that the sliding variable under the FixTC controller can rapidly converge and stabilize approaching zero without oscillating. Although the sliding mode surface of FiniteTC can also rapidly converge to near-zero, there are oscillations at a later stage, which lead to oscillatory control input. Figure 10 depicts the control input variations exhibited by the three controllers. In the initial stage, the control torque of the three controllers is large, among which the PID controller exhibits the largest control torque and the FiniteTC controller exhibits the smallest control torque. When the system is stabilized, the control input under the FixTC controller is relatively small. In the whole control process, the control torques of the PID controller and FixTC controller do not exhibit oscillatory behavior, whereas the control torque under the FiniteTC controller exhibits a large oscillatory phenomenon. This observation indicates that introducing a sign function in the sliding mode controller can easily lead to oscillations in the control input; however, the super-twisting second-order SMC can effectively mitigate the oscillations occasioned by the sign function.

The control performance comparison of the three controllers is illustrated in Table 3.

From the preceding table, we can observe the total control effort in the form of energy consumption for the three controllers. Although the control effort is almost the same, there is a slight difference among the three controllers (i.e., FixTC, PID, and Finite TC). The time of convergence for the proposed controller is short compared to PID and Finite TC. Moreover, the proposed controller exhibits higher accuracy (up to 10^−10^ regarding tracking error as depicted in Table 3).

**Figure 11:**
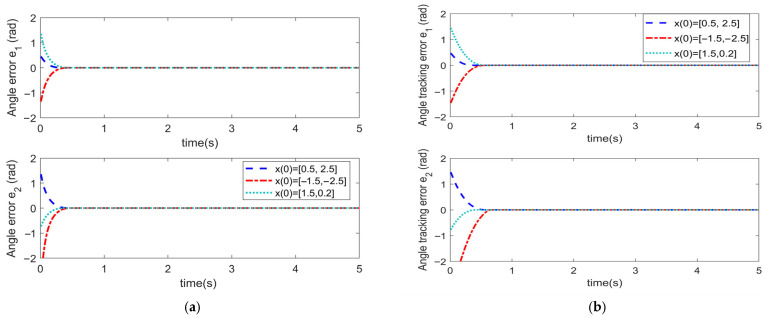
Angle tracking error for the (**a**) FixTC controller and (**b**) FiniteTC controller.

**Figure 12:**
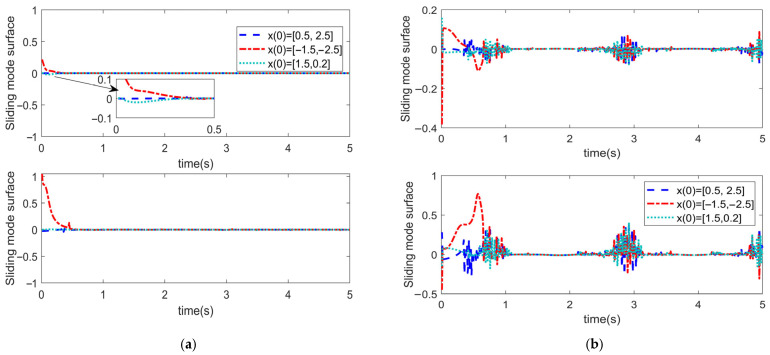
Sliding mode surface for the (**a**) FixTC controller and (**b**) FiniteTC controller.

**Figure 13:**
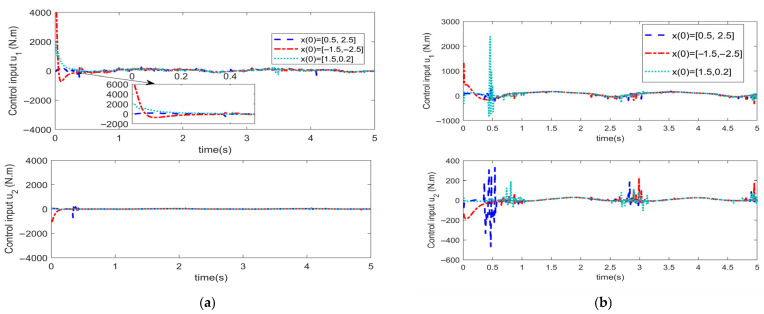
Control input for the (**a**) FixTC controller and (**b**) FiniteTC controller.

To analyze the influence of the initial angle value on the convergence time pertaining to the trajectory tracking error of the manipulator, the initial angle of the manipulator has been set to $x\left(0\right)=\;[$0.5, 2.5]^T^, $x\left(0\right)=\;[$−1.5, −2.5]^T^, and $x\left(0\right)=\;[$1.5, 0.2]^T^, respectively. Numerical simulation has been conducted for the FiniteTC controller with finite-time convergence and the FixTC controller with fixed-time convergence. The parameter settings of each controller remained unchanged. The obtained corresponding simulation results are depicted in Figure 11, Figure 12 and Figure 13.

Figure 11 illustrates the comparison of angle tracking errors for the two controllers. Figure 11a indicates that in the case of the FixTC controller, the convergence time of trajectory tracking errors under the three different initial values hardly changes, which reveals that the convergence time of STSMC with fixed-time convergence is not affected by the initial value of system states. However, for the FiniteTC controller (Figure 11b), there are certain differences in the convergence time of trajectory tracking errors; thus, the convergence time is easily affected by the initial value of the system states.

Figure 12 depicts the convergence trend diagram of the sliding mode surface for the two aforementioned controllers. It is indicated that the convergence time of the sliding variable based on the FixTC controller is almost the same with different initial conditions (Figure 11a), whereas the convergence time of the sliding variable in the case of FiniteTC control is apparently different with various initial conditions, thus demonstrating large oscillations.

Figure 13 depicts the control input for both controllers. Using comparative analysis, it can be observed that initially different initial values of states lead to different oscillations in the control inputs for both controllers. However, after the system is stabilized, the FixTC controller exhibits almost no oscillations, whereas the FiniteTC controller exhibits large oscillations.

## 6. Conclusions

In this paper, the trajectory tracking control problem of a multi-input multi-output (MIMO) manipulator system subject to parameter perturbations and external disturbances was investigated. By integrating the concepts of Immersion and Invariance (I&I) theory and fixed-time convergence theory, a fixed-time super-twisting sliding mode control (ST-SMC) strategy was developed. The proposed method exhibits salient features such as fast convergence, high tracking accuracy, and strong robustness against system uncertainties.

Based on the I&I framework, a disturbance observer was designed for the manipulator dynamics. It was theoretically proven that the proposed observer guarantees exponential convergence of the disturbance estimation error to zero. The disturbance estimation dynamics effectively transform the pure integral action into a proportional–integral adjustment, thereby enhancing both the adaptability and estimation accuracy of the observer.

Furthermore, the fixed-time sliding mode control scheme previously applied to single-input single-output (SISO) systems was extended to MIMO manipulators. A sliding surface with fixed-time convergence characteristics was constructed, and a corresponding super-twisting controller was designed. This controller not only suppresses the inherent oscillations in conventional SMC but also significantly improves the convergence rate and tracking precision of the manipulator’s joint angles.

Theoretical analysis and simulation results demonstrate that, even in the presence of parameter perturbations and external disturbances, the proposed controller achieves robust end-effector trajectory tracking with ultra-high accuracy. Importantly, the convergence time of the tracking error is independent of the initial joint states, making it both predictable and controllable.

## Figures and Tables

**Table 1:** Parameters representing the manipulator and simulation settings.

Symbol	Description	Value	Unit
$m_{1}$	Mass of manipulator	10	kg
$m_{2}$	Mass of manipulator	5	kg
*I*_1_	moment of inertia	0.83	kg·m^2^
*I*_2_	moment of inertia	0.83	kg·m^2^
*l*_1_	joint length	1	m
*l*_2_	joint length	0.5	m
*l*_c1_	joint length	0.5	m
*l*_c2_	joint length	0.25	m
g	gravitational acceleration	9.81	kg·m^2^
$∆m_{1}$	Mass perturbation	0.5sin(t)	kg
$∆m_{2}$	Mass perturbation	0.25sin(t)	kg
$∆I_{1}$	Inertia moment perturbation	0.083sin(t)	kg·m^2^
$∆I_{1}$	Inertia moment perturbation	0.03cos(t)	kg·m^2^
*d*_1_(t)	External disturbance	3sin(0.1 t)	N·m
*d*_2_(t)	External disturbance	(1-exp(−0.1 t))	N·m

**Table 2:** Controller parameters.

Category	Symbol	Value
I&I disturbance observer	$k_{I\&I}$	10
Sliding mode surface	$k_{e1}$	5
	$k_{e2}$	5
	$p_{1}$	5
	$q_{1}$	3
	$p_{2}$	5
	$q_{2}$	3
Super-twisting sliding mode control	$k_{s1}$	$\left[\begin{array}{cc} 500 & 0\\ 0 & 500 \end{array}\right]$
	$k_{s2}$	$\left[\begin{array}{cc} 500 & 0\\ 0 & 500 \end{array}\right]$
	$\gamma_{s1}$	0.5
	$\gamma_{s2}$	1
	$\gamma_{s3}$	1
	$\alpha_{1}$	0.7
	$\alpha_{2}$	1.6

**Table 3:** Control quality indicators.

	Angle Convergence Time		Angle Tracking Accuracy		Total Energy Consumption	
	e1	e2	e1	e2	u1	u2
FixTC	0.42 s	0.40 s	$2\times 10^{-10}$	$2\times 10^{-9}$	$3.3792\times 10^{4}$	$0.3258\times 10^{4}$
PID	0.44 s	0.55 s	$4\times 10^{-2}$	$1\times 10^{-3}$	$3.9479\times 10^{4}$	$0.2944\times 10^{4}$
Finite TC	0.55 s	0.43 s	$2\times 10^{-7}$	$2\times 10^{-5}$	$3.9005\times 10^{4}$	$0.3322\times 10^{4}$

## Data Availability

Data will be made available on request.
